# Point-of-care examination of visual evoked potentials in acutely hospitalized schizophrenia patients

**DOI:** 10.1192/j.eurpsy.2026.10731

**Published:** 2026-07-31

**Authors:** J. Hubenak, L. Miščík Ramešová

**Affiliations:** 1 Department of Psychiatry, University Hospital Hradec Králové; 2 Department of Pathological Physiology, Charles University Faculty of medicine in Hradec Králové, Hradec Králové, Czech Republic

## Abstract

**Introduction:**

Research on visual evoked potentials (VEP) offers the possibility of finding electrophysiological markers of schizophrenia (SCH). VEP research has so far been limited to specialized laboratories. The development of a new portable device for the VEP measurement has brought the possibility of examining acutely ill patients. Previous research on VEP in SCH has shown a decrease in the amplitude of the P300 wave and a prolongation of the P300 latency as a promising endophenotype of SCH.

**Objectives:**

The aim was to measure VEP in hospitalized SCH patients and verify whether previously P300 connected findings also apply in this population. The mobile VEP device also allows for the determination of the speed of the motor response (i.e. reaction time, RT) to a visual stimulus. The secondary aim was to evaluate the relationship between physical parameters and immunological indicators (i.e. ratios of blood element counts from the blood count) with VEP values.

**Methods:**

VEPs were measured in SCH patients hospitalized at the Dept. of Psychiatry of the Univ Hos in Hradec Králové. VEPs were also obtained from age and sex matched controls. The amplitude and latency of the P300 were evaluated. The motor response (button press) RT to a visual stimulus was measured. Other obtained values were: Body Mass Index (BMI), Positive and Negative Syndrome Scale (PANSS), Brief negative Symptom Scale (BNSS), Clinical Global Impressions-Severity (CGI-S) scales, B-CATS neuropsychological battery (containing tests TMT-A, TMT-B, Digit symbol, Animal naming), blood count.

**Results:**

Data from 60 patients and 60 healthy controls (HC) have been obtained so far. The P300 had a (p = 0.18) reduced amplitude, prolonged latency, and RT in SCH (both p < 0.001). RT increased faster (slowed down) in SCH in relation to age compared to HC. Moderate and weak correlations (Spearman’s rho) were achieved between cognitive test scores and VEP values. BMI was only weakly correlated with P300 amplitude, but not significantly correlated with other VEP values. Blood counts were also not significantly correlated with VEP values. BNSS and PANSS total scores both achieved moderate correlations with RT and with the difference in RT and latency. Olanzapine medication equivalent achieved only weak correlations with VEP. Correlations of VEP with CGI-S were only weak.

**Image 1:**
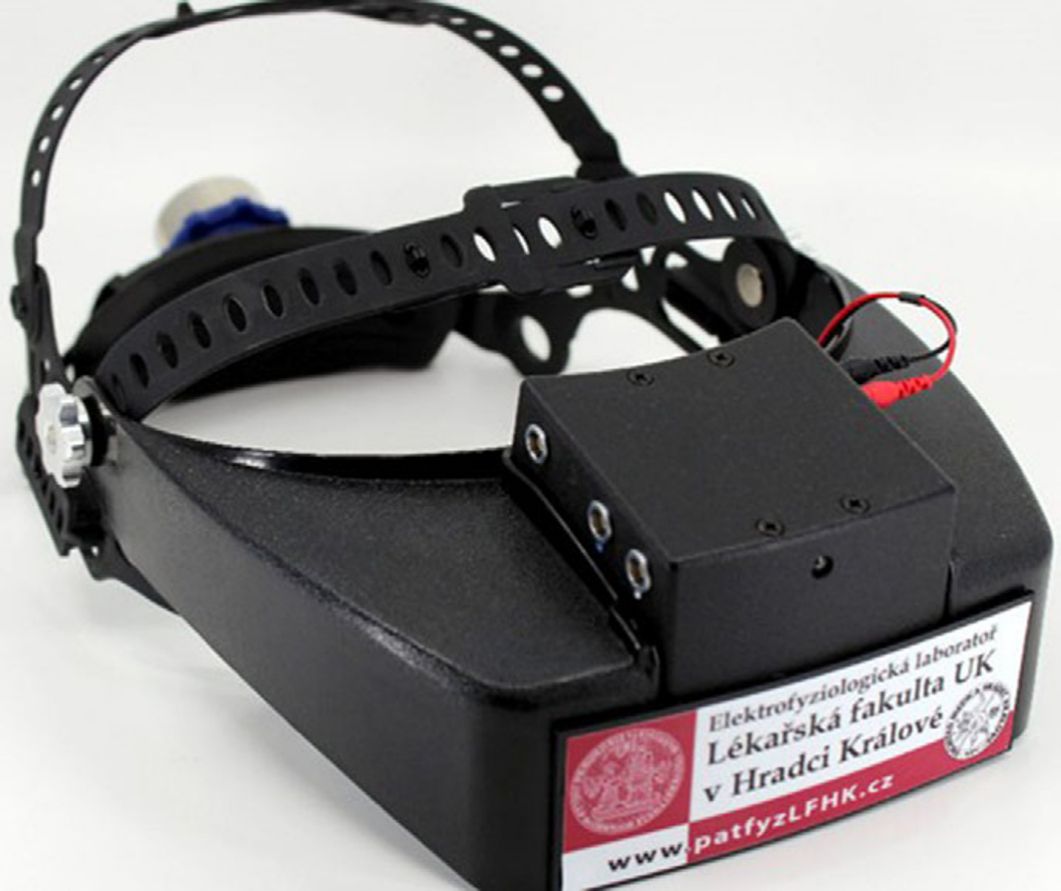

**Image 2:**
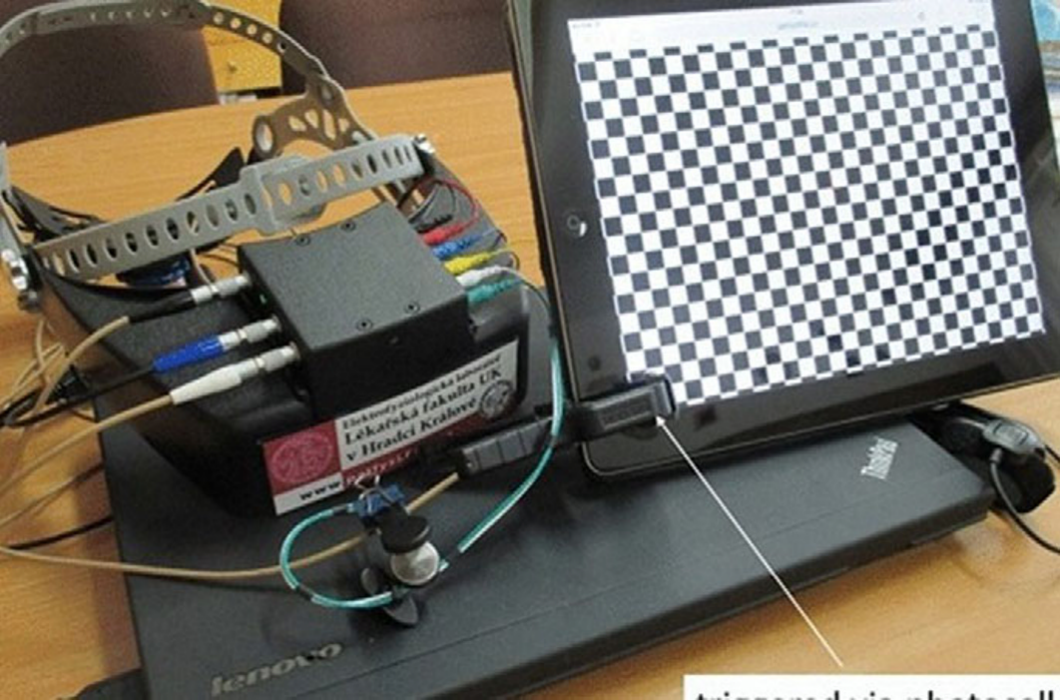
Image 2: Long description.

**Image 3:**
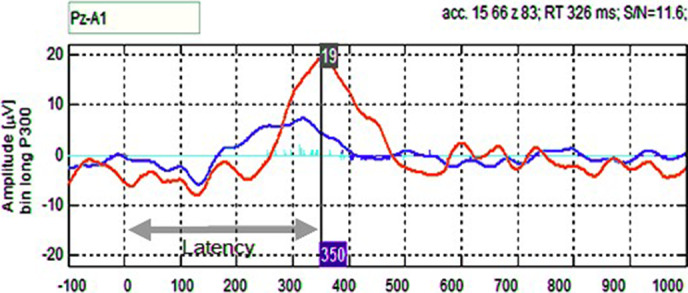
Image 3: Long description.

**Conclusions:**

The faster RT slowing with increasing age in SCH compared to HC is a new finding. This may indicate faster brain aging in SCH. VEP values are apparently not distorted by BMI or blood count values. The achievement of moderate correlations with some cognitive tests, PANSS and BNSS values suggest the possibility of using VEP for quantifying both positive and negative and even symptoms of cognitive dysfunction in SCH. A deeper analysis of VEP recordings is needed to find more significantly correlating electrophysiological parameters compared to the currently used P300.

**Disclosure of Interest:**

None Declared

## Long descriptions

### Image 2: Long description

On the left, a head-mounted apparatus consists of a grey adjustable plastic frame and a black E E G amplifier box. Multiple colored wires (red, blue, yellow, white, and green) are plugged into the side of the amplifier. The amplifier sits on a black Lenovo laptop. A label on the amplifier reads Elektrofyziologicka laborator Lekarska fakulta U K v Hradci Kralove.

On the right, a tablet is propped up on the laptop, displaying a high-contrast black and white checkerboard pattern. A small black sensor is clipped to the bottom left corner of the tablet screen. A white line points from a text box at the bottom right that reads triggered via photocell to this sensor, indicating the synchronization mechanism between the visual stimulus and the recording device.

Navigate back to Image 2:.

### Image 3: Long description

A line graph with a horizontal x-axis representing time in milliseconds from negative 100 to 1000 and a vertical y-axis representing Amplitude in microvolts from negative 200 to 20. The y-axis label is Amplitude mu V bin long P 300. Top right metadata reads acc. 15 66 z 83 R T 326 ms S forward slash N equals 11.6. A top left box contains P z minus A 1.

Two primary waveforms are plotted:

* A red line shows a significant positive peak reaching approximately 19 microvolts at the 350 millisecond mark. This peak is highlighted by a vertical black line with a grey box at the top containing the number 19 and a purple box at the bottom containing the number 350.

* A blue line follows a similar but lower-amplitude path, peaking at approximately 8 microvolts around 300 milliseconds before returning to near zero.

A horizontal double-headed grey arrow spans from 0 to 350 milliseconds on the x-axis, labeled Latency in green text. Both waveforms show minor oscillations before 0 milliseconds and after 500 milliseconds, fluctuating between negative 5 and positive 5 microvolts.

Navigate back to Image 3:.

